# Intellectual disability and autism spectrum disorders ‘on the fly’: insights from *Drosophila*

**DOI:** 10.1242/dmm.039180

**Published:** 2019-05-13

**Authors:** Mireia Coll-Tané, Alina Krebbers, Anna Castells-Nobau, Christiane Zweier, Annette Schenck

**Affiliations:** 1 Department of Human Genetics, Donders Institute for Brain, Cognition and Behaviour, Radboud University Medical Center, 6525 GA Nijmegen, The Netherlands; 2 Institute of Human Genetics, Friedrich-Alexander-Universität Erlangen-Nürnberg, 91054 Erlangen, Germany

**Keywords:** Neurodevelopment, ASD, ID, *Drosophila*, Fruit fly, Brain

## Abstract

Intellectual disability (ID) and autism spectrum disorders (ASD) are frequently co-occurring neurodevelopmental disorders and affect 2-3% of the population. Rapid advances in exome and genome sequencing have increased the number of known implicated genes by threefold, to more than a thousand. The main challenges in the field are now to understand the various pathomechanisms associated with this bewildering number of genetic disorders, to identify new genes and to establish causality of variants in still-undiagnosed cases, and to work towards causal treatment options that so far are available only for a few metabolic conditions. To meet these challenges, the research community needs highly efficient model systems. With an increasing number of relevant assays and rapidly developing novel methodologies, the fruit fly *Drosophila melanogaster* is ideally positioned to change gear in ID and ASD research. The aim of this Review is to summarize some of the exciting work that already has drawn attention to *Drosophila* as a model for these disorders. We highlight well-established ID- and ASD-relevant fly phenotypes at the (sub)cellular, brain and behavioral levels, and discuss strategies of how this extraordinarily efficient and versatile model can contribute to ‘next generation’ medical genomics and to a better understanding of these disorders.

## Abstract (precis)

**Summary:** This Review summarizes some of the exciting work that has used *Drosophila* as a model for the ever-expanding field of intellectual disability and autism spectrum disorders. It highlights disease-relevant assays, emerging themes and future challenges.

## Introduction

Intellectual disability (ID) and autism spectrum disorders (ASD) are major neurodevelopmental disorders with a frequency of 2-3% in western countries (Bourke et al., 2016). ID is defined by significant limitations in both intellectual functioning and adaptive behavior before the age of 18 years, and is usually reflected by an IQ below 70 (Ropers, 2010). ASD is a collective term for a spectrum of behavioral phenotypes including deficits in communication and social interaction, and restricted and repetitive behaviors, interests and activities. ID and ASD often co-occur, with an estimated 10% of children with ID having autistic symptoms and with 70% of individuals with autism also having ID (Oeseburg et al., 2011; Schwartz and Neri, 2012).

Because of their frequency and lifelong nature, ID and ASD are an immense socioeconomic burden for the affected families and for healthcare systems. They represent a large unsolved problem in modern medicine due to limited treatability, partially caused by their poorly understood biology. Most ID cases are monogenic, meaning that mutations in a single gene are sufficient to lead to the disorder. Inheritance patterns, such as sporadic *de novo* mutations or homozygosity in consanguineous families (Deciphering Developmental Disorders Study, 2017; Najmabadi et al., 2011), facilitate disease gene and variant identification (Vissers et al., 2016). So far, little is known about oligogenic inheritance (see Box 1 for a glossary of terms) in ID and the identity of modifiers contributing to a large clinical variability and incomplete penetrance in some cases. In contrast, ASD often represent a genetically complex disorder with oligogenic or polygenic causes, including a combination of both rare *de novo* variants and more common inherited variants (Chaste et al., 2017). This complex genetic architecture hampers the identification of high-confidence risk-conferring ASD genes. However, this is mainly true for the subset of ‘high-functioning’ ASD cases, who have normal cognitive function. ASD in combination with ID is often monogenic (Arnett et al., 2018). Owing to this large clinical and molecular overlap, monogenic causes of ID also provide us with an unique molecular window into the biology and (patho)mechanisms of ASD.

**Box 1. Glossary**

**Angelman syndrome (OMIM #105830):** neurodevelopmental disorder characterized by intellectual disability (ID), typical abnormal behaviors, movement or balance problems, and severe speech and language impairments. Around 75% of cases are caused by *de novo* deletions in 15q11.2-q13 on the maternal chromosome 15. The remaining cases are because of paternal uniparental disomy 15, point mutations in the UBE3A gene or rare imprinting defects (Buiting et al., 2016).

**Arborization pattern:** tree-like morphological arrangement of dendritic branches.

**Basal ganglia:** group of subcortical nuclei (neuronal population) in the vertebrate brain that play a critical role in motor control and cognition (e.g. in reward-based learning).

**Boutons:** round-shaped varicosities of the neuromuscular junction (NMJ) presynaptic terminal that house active zones (the neurotransmitter release machinery).

**Central complex:** a set of neuropil-rich structures (protocerebral bridge, fan-shaped body and ellipsoid body) that integrate complex sensorial (environmental) information with the fly's internal state and previous experience into an appropriate behavioral response (shaped as a motor output) (Wolff and Rubin, 2018).

**Dendritic arborization (da) sensory neurons:** nociceptive dopaminergic neurons present in the larval body wall.

**Dendritic spine:** postsynaptic compartment protruding from dendrites, receiving input from a single synapse (axon terminal).

**Electroretinogram:** *Drosophila* eye voltage recording reflecting retinal electrical activity upon light stimulation (Ugur et al., 2016).

**Fragile X syndrome (OMIM # 300624):** most common monogenic cause of ID and ASD, caused by CGG-repeat expansion (>200) in the 5′ untranslated region (5′-UTR) of the *FMR1* gene.

**Giant-fiber system (recordings):** neural circuit controlling escape-response behavior in adult *Drosophila*. Electrophysiological recordings can be performed through the direct stimulation of the giant fiber neurons and recording from their output muscles (Allen and Godenschwege, 2010).

**Inborn errors of metabolism:** genetic disorders causing specific metabolic defects due to mutations in genes encoding metabolic enzymes or transporters.

**Light-off jump habituation:** paradigm used to assess non-associative learning habituation. Repeated light-off stimuli generate an initial jump (startle reflex) response that gradually diminishes due to a learned adaptation to the stimuli, not due to sensory desensitization or motor fatigue.

**Non-declarative memory:** implicit memory acquired and used without conscious awareness. A classic example is motor memory.

**Oligogenic inheritance:** trait modulated by a small number of genes or loci (Badano and Katsanis, 2002).

**Purkinje cell:** large GABAergic neurons in the cerebellar cortex that regulate and coordinate motor function.

**Non-REM and REM sleep:** the two main components of sleep. REM stands for and is characterized by rapid eye movement, and by low-amplitude and mixed-frequency waves on electroencephalogram (EEG). In contrast, non-REM sleep shows mainly slow wave activity on EEG.

**Rett syndrome (OMIM #312750):** neurodevelopmental disorder characterized by an arrest in development before the second year of life and a regression of all acquired skills; patients present with ID, loss of speech, stereotypic hand movements, microcephaly and seizures. Rett syndrome occurs almost exclusively in females, and is caused by mutations in the *MECP2* gene (Amir et al., 1999).

**Suprachiasmatic nucleus:** principal circadian pacemaker of the mammalian brain located in the hippocampus.

**T2A-Gal4:** a cassette that disrupts the gene into which it is integrated and at the same time permits Gal4-mediated induction of *UAS* alleles under the gene's endogenous regulatory elements (Diao et al., 2015).

**Whole-exome sequencing:** genomic technique to investigate all protein-coding regions of the genome (exome).

The development of new tools, such as next-generation sequencing, has brought substantial progress in ID/ASD gene and variant identification (Sanders, 2018; Vissers et al., 2016). Genetically, both ID and ASD are extremely heterogeneous, with more than 1150 confirmed disease-associated genes (Kochinke et al., 2016; SysID database, updated on October 2018, https://sysid.cmbi.umcn.nl/). Within this large group, molecular pathways and networks emerge, linking variants with overlapping phenotypes (Kochinke et al., 2016). However, as chromosomal microarray analysis currently identifies ca. 20% (Miller et al., 2010) and (trio) whole-exome sequencing (Box 1) ca. 40% (Deciphering Developmental Disorders Study, 2017) of causative aberrations, a significant fraction of ID and the majority of ASD patients remain without a genetic diagnosis.

Although current treatment options are limited to a small number of ID/ASD disorders deriving from metabolic deficits [inborn errors of metabolism (Box 1)] (van Karnebeek and Stockler, 2012), this does not necessarily mean that opportunities to improve cognitive impairments and associated behavioral problems are non-existent. Generalizations, such as deeming ID and ASD as barely reversible based on their early onset and classification as neurodevelopmental disorders, might hinder efforts to identify effective treatment for specific conditions. In fact, still very little is known about the degree of developmental versus postnatal (acute lack of a required gene/protein function) contribution to brain dysfunction in most ID and ASD disorders, i.e. it is unclear to what extent the brain is not functioning because it has wrongly ‘hardwired' during development and to what extent because an important component for postnatal functioning is acutely missing. In the past years, several studies have provided impressive examples of how impaired gene/protein function can be restored in adult animals (Guo et al., 2000; Guy et al., 2007; Kramer et al., 2011; Lee et al., 2014; McBride et al., 2005). These findings raise hope that cognitive impairment in several forms of ID and ASD can be reversed or mitigated.

In summary, ID and ASD are dynamic fields of research with a number of big challenges ahead, including the identification of additional disease genes to allow better diagnostics, the characterization of candidate genes to better understand the neurobiology of the associated disorders, and the development of successful treatment approaches. Model organisms are widely used in the endeavor to overcome these bottlenecks. *Drosophila melanogaster*, the fruit fly, is a well-established genetic model, and highly suited to study the nervous system from genes to behavior (Ugur et al., 2016). In general, *Drosophila* is a cheap, genetically highly accessible, and, compared to vertebrates, a rather simple organism with high potential for both in-depth and high-throughput research.

The aim of this Review is to summarize some of the exciting work that has already drawn attention to *Drosophila* as a model for ID and ASD. We highlight disease-relevant fly phenotypes at the morphological, functional and behavioral levels, and discuss the future challenges in medical genomics that could be met by this extraordinarily efficient and versatile model.

## Using *Drosophila* to overcome bottlenecks in ID and ASD research: relevant features and paradigms

**Fig. 1.**
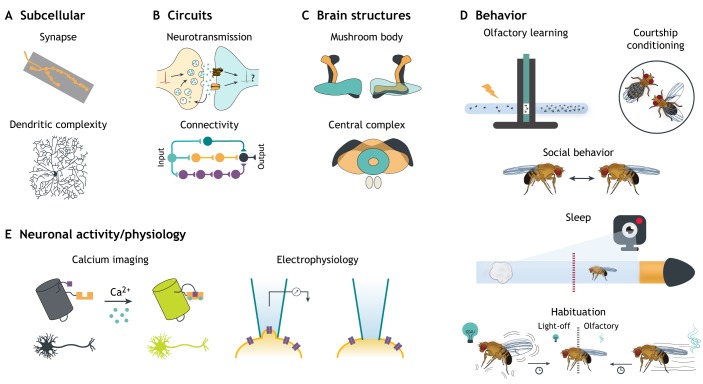
**Modeling ID and ASD in *Drosophila* – from (sub)cellular defects to aberrant behavior.** This figure summarizes the commonly utilized ID- and ASD-relevant phenotype assays at various levels of complexity: from subcellular and circuit-level to brain structures, neuronal activity and behavior. (A) At the subcellular level, an NMJ and a type-IV da neuron with its complex dendritic tree serve as models to assess synapse morphology and dendritic complexity, respectively. (B) Circuits can be studied at the functional or connectivity level. Top: a synaptic cartoon with ongoing neurotransmission, with neurotransmitter release from the presynaptic terminal into the synaptic cleft and subsequent binding to receptors present in the postsynaptic terminal. Bottom: a hypothetical circuit, which is a parallel after-discharge circuit: an input neuron discharges to different chains of neurons, each one with a different number of synapses, and eventually all converge onto a single output neuron. (C) Many neuroanatomical entities can be studied in *Drosophila*, and the mushroom body (MB) and the central complex (CC) are of particular interest for ID and ASD modeling (see text). (D) Many behavioral assays can be used to assess ID- and ASD-relevant readouts. At the top of the panel, the two most widely used assays to assess associative learning and memory are depicted: olfactory learning, as conducted with a T-maze in which an electric shock is used as a negative stimulus, and courtship conditioning, with a naïve male courting a pre-mated female. Social behavior in *Drosophila* can be assessed, for instance, through the study of intra-fly distance. Sleep has been classically studied in the fly with single-beam activity monitors (red dashed line), but video tracking is increasingly being used. Lastly, non-associative learning is studied in *Drosophila* in light-off or olfactory habituation learning paradigms. Initial responses to these cues gradually wane. (E) Neuronal activity/physiology levels can be assessed by Ca^2+^ imaging (left) using genetically encoded Ca^2+^ indicators and by electrophysiological recordings, such as patch-clamp (right).

With the advent of exome sequencing, the major bottleneck in ID changed from gene identification to understanding gene function, interpreting the effect of the variants found in patients, and understanding various pathomechanisms. About three-quarters of all ID genes identified are conserved in *Drosophila* (Oortveld et al., 2013; Vissers et al., 2016). Despite the low conservation of the central nervous system (CNS) anatomy between flies and humans, ID-relevant biological processes are highly conserved at the molecular, cellular and synaptic level (Tian et al., 2017). While *Drosophila* research has so far focused on modeling ID rather than ASD, their genetic and clinical overlap makes the potential of such studies obvious. In Fig. 1, we have summarized the most widely used assays and systems to study the hallmarks and underlying mechanisms of ID and ASD in *Drosophila*.

### Neuromuscular junction as a model synapse

A significant number of ID/ASD genes are required for synaptic transmission (Srivastava and Schwartz, 2014) and/or synaptic organization, which may directly contribute to the synaptic morphology defects found in postmortem studies and various animal models (reviewed in Varghese et al., 2017). The *Drosophila* larval neuromuscular junction (NMJ) has been used for decades to investigate synapse morphology, development and neurotransmission in fundamental and disease model studies (Fig. 1A). The structural characteristics of NMJs make them an ideal model: they are relatively large and readily accessible, and thus suitable for electrophysiological and morphological investigation (Frank, 2014; Nijhof et al., 2016). However, the NMJ is peripheral and connects to a muscle instead of a postsynaptic neuron; therefore, some processes that operate at NMJs can differ from those at CNS synapses. Despite this, *Drosophila* NMJs share many features with vertebrate CNS synapses. For instance, they are glutamatergic, like the majority of excitatory synapses in the mammalian brain. The presynaptic component is composed of boutons (Box 1). The opposing postsynaptic membrane contains ionotropic glutamate receptors as well as postsynaptic signaling complexes, assembled in the postsynaptic density (Harris and Littleton, 2015). Pre- and postsynaptic molecular machineries include many highly conserved key regulatory proteins involved in ID and ASD, such as neurexins, synapsin I, synaptotagmins, ionotropic glutamate receptors (e.g. GRIN2A, GRIN2B and GRIK2) and PSD-95 (Dlg in *Drosophila*) (Han et al., 2015; Harris and Littleton, 2015). Similarities also extend to conserved processes regulating fundamental synaptic features, including synaptic plasticity, homeostasis, development and neurotransmitter recycling (Menon et al., 2013). Recent work in *Drosophila* has unraveled novel synaptic functions of classic ID/ASD genes. For instance, the fly NMJ was key in identifying presynaptic roles of proteins traditionally thought of as being only postsynaptic. These include *Shank*, the unique ortholog of human *SHANK1-SHANK3*, implicated in ASD and other neuropsychiatric conditions (Harris et al., 2016; Wu et al., 2017), and *Dnlg4* (*NLGN4* ortholog), a member of the neuroligin family, several of which are implicated in ID/ASD (Zhang et al., 2017).

### Multidendritic neurons as a model for dendrites

Changes in dendritic architecture have long been reported in various neurodevelopmental conditions (Kaufmann and Moser, 2000; Kulkarni and Firestein, 2012). The first histological studies of ID patients' brains back in the 1970s showed a reduced complexity of the arborization pattern (Box 1) of their dendrites, and an increased number of immature dendritic spines (Box 1) (Purpura, 1975). Similar findings have been reported in Rett syndrome (Box 1) and other forms of ID/ASD, e.g. ID/ASD associated with mutations in *CAMK2A*, *SHANK3* or *IL1RAPL1* (Pardo and Eberhart, 2007; Stephenson et al., 2017).

A well-established model to study dendritic tree morphology in *Drosophila* are the dendritic arborization (da) sensory neurons (Box 1) of the peripheral nervous system. Depending on their morphology and function, four different classes of da neurons can be defined (I-IV). Type-IV da neurons display the most complex arborization, and tile the complete body wall with minimum overlap between neighboring neurons (Corty et al., 2009; Jan and Jan, 2010; Fig. 1A). Owing to this, as well as their location in the larval body wall and their planar nature, they are easy to identify, access, trace and quantify. Moreover, like NMJs, they can also be imaged *in vivo* over time (Jan and Jan, 2010; Satoh et al., 2012).  The da neurons have a well-characterized and stereotyped architecture, which is achieved through a strict regulation of genetic programs and molecular pathways (Corty et al., 2009; Gao et al., 1999; Jan and Jan, 2010; Tassetto and Gao, 2006). One limitation of using da neurons as a dendritic model is that these, and most other *Drosophila* neurons, lack dendritic spines.

Taking advantage of this approach, researchers have uncovered the role of multiple ID/ASD genes and pathways in dendrite development. These include the gene *DYRK1A* (*minibrain* in *Drosophila*), gain of which is associated with Down syndrome (Altafaj et al., 2001; Guimera et al., 1996), whereas heterozygous disruption of the gene causes ID, ASD and microcephaly (Møller et al., 2008; O'Roak et al., 2012; van Bon et al., 2011). Using da neurons as a model, Ori-McKenney et al. found that altering Minibrain levels disrupts dendrite morphology and neuronal physiology due to abnormal phosphorylation of β-tubulin, a direct Minibrain substrate, which results in inhibited tubulin polymerization (Ori-McKenney et al., 2016). Additionally, several upstream (e.g. Wnt5) and downstream (e.g. Trio and Rho1) effectors of the Wnt pathway, implicated in the etiology and pathophysiology of many ID and ASD disorders (Kwan et al., 2016; Vorstman et al., 2017), were also recently uncovered to be critical in dendrite termination and delimitation of dendritic boundaries in *Drosophila* (Yasunaga et al., 2015).

### Neuronal activity assessed by electrophysiology and calcium imaging

It appears likely that the above-mentioned morphological anomalies in ID and ASD correlate with anomalies in neuronal activity. Indeed, altered neuronal activity, measurable by non-invasive methods, has been reported in patients (Carter Leno et al., 2018; Guy et al., 2007; Knoth et al., 2018), as well as in some *in vitro* models, such as cortical neuron cultures and induced pluripotent stem cells (Griesi-Oliveira et al., 2015; Martens et al., 2016). The manipulable nature and reduced complexity of the *Drosophila* brain allows in-depth assessment of neuronal function, from a single cell to the whole network (Fig. 1E). In this context, electrophysiological assays from patch-clamp (Murthy and Turner, 2013) to whole-brain (van Swinderen and Greenspan, 2003) recordings, as well as electroretinograms (Box 1), NMJ electrophysiology and giant-fiber-system recordings (Box 1), have proven to be informative tools to assess neuronal activity (Ugur et al., 2016).

Several of these electrophysiological measurements can be combined with live imaging of protein or organelle trafficking and calcium (Ca^2+^) imaging, as facilitated by ever-improving genetically encoded calcium indicators (Simpson and Looger, 2018; Yang et al., 2018), to provide insights into the molecular control of neurotransmission. Furthermore, Ca^2+^ imaging can be performed *ex vivo* (Tong et al., 2016) and *in vivo* to simultaneously measure activity and behavior in the context of various circuits and developmental stages (Macleod, 2012; Seelig et al., 2010).

### Mushroom body

Deficits in learning and memory are one of the main hallmarks of ID (Detterman, 1987; Vicari, 2004). Moreover, children with ASD also show impaired memory for complex information and poor working memory for spatial information (Williams et al., 2006). *Drosophila* has been widely used to investigate learning and memory. Before discussing behavioral paradigms used for learning and memory assessment in the next section, we will briefly describe the brain areas important for learning, and memory formation and consolidation. One of the key mammalian brain centers involved in several forms of learning and memory is the hippocampus (Moser et al., 2008; Squire, 1992; Winocur, 1990). Numerous ID and ASD genes have been shown to be important for hippocampal development and function, including genes involved in epigenetic remodeling (Lagali et al., 2010), neuronal migration and differentiation (Kepa et al., 2017; Wegiel et al., 2010), or synaptic circuitry maturation (Lanore et al., 2012; Roussignol et al., 2005).

Although structurally very different from the mammalian brain, some *Drosophila* brain centers have been argued to have analogy with human brain structures in terms of neuronal connectivity and behavioral output. The mushroom body (MB) is often referred to as the brain structure analogous to the mammalian hippocampus, as it has been widely implicated in insect learning and memory (Campbell and Turner, 2010; Heisenberg et al., 1985). It has also been proposed as an analog to both the cerebellum and the cortex due to a similar architecture and gene expression, respectively (Farris, 2011; Tomer et al., 2010). Interestingly, although the cerebellum has classically been associated with motor function, there is increasing evidence for its role in cognition (Leiner et al., 1993; Vandervert, 2016) and as a key region in ASD susceptibility (Chen et al., 2017; Peter et al., 2016; Wang et al., 2014). This association has, however, been attributed to dysfunction of Purkinje cells (Box 1) (Clifford et al., 2019; Tsai et al., 2012), for which no correlate has been identified in *Drosophila*, thus limiting studies into this interesting topic.

The MB is a neuropil-rich structure composed of ∼2500 Kenyon cell axons. These neurons receive and integrate inputs from several sensory pathways, including olfactory, gustatory, visual and auditory (Masek and Scott, 2010; Vogt et al., 2014) information that can be modified by reward or punishment via dopaminergic input (Liu et al., 2012; Riemensperger et al., 2005; Fig. 1C). MB output is glutamatergic, GABAergic or cholinergic (Aso et al., 2014) and is carried to convergent brain areas, ultimately resulting in modified behavior. MBs have been studied mainly for their role in associative learning. However, they are also involved in other behaviors, such as olfactory learning (Heisenberg et al., 1985), habituation (Acevedo et al., 2007; Glanzman, 2011), sleep (Joiner et al., 2006; Sitaraman et al., 2015), context generalization (Liu et al., 1999), habit formation (Brembs, 2009), temperature preference (Bang et al., 2011; Hong et al., 2008) and, recently, perceptual decision-making (DasGupta et al., 2014; Groschner et al., 2018). Some of these behaviors are highly relevant for ID and ASD, as will be discussed further in this Review. The MB is thus a very attractive system to link disease genes to their cellular function and disease-relevant behavior, and thus to a better understanding of disease pathology.

### Associative learning and memory

The most commonly used assay to investigate learning and memory in *Drosophila* is olfactory classical conditioning (Fig. 1D). In this paradigm, odors (the conditional stimulus) are coupled to either a positive (e.g. sugar reward) or negative (e.g. electric shock) stimulus (the unconditioned stimulus). Upon successful learning, the flies will either avoid or prefer the associated odor even in the absence of the unconditional stimulus (Busto et al., 2010; Quinn et al., 1974). Another widely used approach to assess associative learning is courtship conditioning. This paradigm is based on the reduction of male courtship behavior in response to sexual rejection of a non-receptive pre-mated female (Siegel and Hall, 1979). Changes in courtship behavior can be easily scored by assessing the stereotyped pattern of behavior in males (summarized in Spieth, 1974). Learning, and short- and long-term memory can be assessed with both olfactory and courtship conditioning paradigms (Busto et al., 2010; Quinn et al., 1974), and both behaviors depend on the MB (de Belle and Heisenberg, 1994; McBride et al., 1999). In *Drosophila*, short-term memory is referred to as the memory present immediately after training. It rapidly decays, within an hour, whereas long-term memory can persist for days (Kahsai and Zars, 2011). An obvious limitation of *Drosophila* is that the established short/long-term memory paradigms probe analogs of non-declarative memory (Box 1; Brem et al., 2013) only.

The groundbreaking contribution of *Drosophila* to our molecular understanding of learning and memory is undebatable. Seymor Benzer and colleagues identified the first learning and memory genes, *dunce* and *rutabaga*, in *Drosophila* (Byers et al., 1981; Dudai et al., 1976; Livingstone et al., 1984). Both genes act in the cyclic AMP (cAMP) pathway, a second messenger activated by G protein-coupled receptor activation and Ca^2+^/Calmodulin. This pathway converges on the cAMP response element-binding protein (CREB) transcription factor to regulate a transcriptional program driving long-term but not short-term memory (Androschuk et al., 2015). Several ID genes have been linked to cAMP signaling, including *CREBBP* (encoding CBP, a CREB co-factor) (Petrif et al., 1995), *FMR1* (Akshoomoff et al., 2015) and *NF1* (Guo et al., 1997). Numerous additional ID/ASD genes converge onto CREB, which also integrates other learning- and memory-related pathways. This includes the Ras-MAPK signaling pathway (Guo et al., 2000; Pagani et al., 2009), which is mutated in a group of ID/ASD disorders referred to as rasopathies (Krab et al., 2008). Recent research into ID/ASD-associated genes highlights the complexity of regulating short- and long-term memory. Unexpectedly, ID genes encoding different subunits of the same protein complex, SWI/SNF, differentially affect MB-encoded short- versus long-term memory (Chubak et al., 2019).

Some ID/ASD gene orthologs have been unbiasedly identified as genes regulating *Drosophila* learning and/or memory, independent of their disease implication, e.g. the *Drosophila* ortholog of *FLNA* (Battaglia et al., 1997), *cheerio* (Dubnau et al., 2003).

### Circadian rhythm and sleep

Many individuals with ID and/or ASD suffer from sleep disturbances (Ballester et al., 2019; Geoffray et al., 2016; van de Wouw et al., 2013; Veatch et al., 2017). A study from 2013 reported 72% of ID patients to have sleep disturbances (van de Wouw et al., 2013), while a more recent study characterized various qualitative components of sleep in ASD patients, and found an increased number of awakenings during the night, sleep onset latency and reduced sleep efficiency (Ballester et al., 2019). Disturbed sleep does not only negatively affect the emotional status and social behavior of patients, but also their cognitive functioning (Geoffray et al., 2016; Veatch et al., 2017).

Some sleep problems can be attributed to defects in the circadian rhythm driven by dysregulation of a highly conserved molecular pacemaker/clock that oscillates in a ∼24 h rhythm and synchronizes physiology and behavior to the time of the day (Dubruille and Emery, 2008). Some ID/ASD patients have a shift in their circadian clock (Ballester et al., 2019; Maaskant et al., 2013). *Drosophila* is an excellent model organism to study the circadian clock and circuit, as supported by the 2017 Nobel Prize in Physiology or Medicine for the discoveries of molecular mechanisms controlling circadian rhythm. In the fly brain, the expression of the pacemaker is restricted to a small set of neurons and glia cells (Zhang et al., 2018), resembling the function of the mammalian suprachiasmatic nucleus (Box 1) (Dubowy and Sehgal, 2017).

*Drosophila* has also delivered fundamental insights into the regulation and function of sleep (Dubowy and Sehgal, 2017; Emery and Reppert, 2004). Sleep in *Drosophila* is defined as five or more minutes of inactivity in which flies show an increased arousal threshold. Circadian behavior and sleep can be measured by assessing locomotor activity (Greenspan et al., 2001), as classically done in the *Drosophila* Activity Monitor (DAM) system (TriKinetics, Waltham, MA, USA). Increasingly used video-tracking-based methods may be more accurate (Garbe et al., 2015) and allow assessment of additional parameters, such as arousal, sleep pressure and feeding [e.g. DART (*Drosophila* ARousal Tracking) system (Faville et al., 2015), ethoscope (Geissmann et al., 2017), ARC (Activity Recording Capillary Feeder) or CAFE (Murphy et al., 2017)]. Moreover, sleep can be modified by stimulants and hypnotics, and is regulated by both the circadian clock and a homeostatic system that determines sleep need, which shows the conserved nature of sleep properties (Shaw et al., 2000). Although there is increasing evidence for dynamic changes in the sleep intensity of *Drosophila* (van Alphen et al., 2013), flies do not display the typical sleep stages described in humans, e.g. non-REM/REM sleep (Box 1). Many brain centers and neuronal clusters have been involved in sleep promotion or inhibition (reviewed in Dubowy and Sehgal, 2017).

When mutated, many *Drosophila* orthologs of human ID and ASD genes have been reported to cause sleep disturbances. Neurexins and neuroligins are key adhesion molecules required for proper synapse formation, homeostasis and function (Dean et al., 2003; Missler et al., 2003). Neurexin 1 in flies regulates nighttime sleep due to its role in mediating synaptic transmission of a subset of MB neurons (Tong et al., 2016), and its loss leads to sleep fragmentation and circadian defects (Larkin et al., 2015). Neurexin receptors, the neuroligins (Nlg proteins), have also been implicated in sleep. *Nlg4* mutant flies display abnormal nighttime sleep due to impaired GABA neurotransmission in clock neurons (Li et al., 2013). This effect on sleep is not exclusive of *Dnlg4*, as has recently been reported for *Dnlg2* (Corthals et al., 2017). Patients with mutations in these genes suffer from sleep disturbances (Harrison et al., 2011; Vaags et al., 2012).

### High potential: central complex, social behavior and habituation learning

The assays discussed above are providing more insights into the pathology of ID and ASD disorders than we are able to acknowledge in this Review. Nevertheless, an increasing amount of novel paradigms have been barely tapped into to investigate ID/ASD but have, we believe, high potential to make significant contributions to the field in the future. In this section, we draw attention to some of these: the *Drosophila* central complex (CC; Box 1), to social behaviors and habituation learning (Fig. 1D).

Increasingly, the literature has pointed to dysfunction in the basal ganglia (Box 1) in ASD and other neuropsychiatric conditions (Riva et al., 2018; Subramanian et al., 2017). This subcortical structure shows homology with the insect CC regarding genetic developmental programs, microarchitecture and regulated behaviors (Lin et al., 2013; Strausfeld and Hirth, 2013). It serves as the integration center for sensory inputs, particularly for space representation and spatial control of motor behavior, and is also involved in various types of memory (Liu et al., 2006; Neuser et al., 2008; Ofstad et al., 2011), arousal and sleep (Donlea et al., 2018, 2011). So far, reports of ID/ASD gene function in the CC are scarce [e.g. *RSK2* (Kuntz et al., 2012; Thran et al., 2013); *SIM2* (Pielage et al., 2002)]. However, given its key role in memory, arousal and sleep, processes highly relevant to ID/ASD (van Alphen and van Swinderen, 2013), it is likely to emerge as a pertinent system to be investigated in *Drosophila* ID/ASD models.

One of the main criteria for diagnosing ASD as stated in the latest Diagnostic and Statistical Manual of Mental Disorders (DSM-5) are ‘persistent deficits in social communication and social interaction across multiple contexts’. These can manifest as a wide variety of deficits: from social-emotional reciprocity, to verbal and nonverbal communicative behaviors needed for social interactions, as well as deficits in establishing and understanding relationships (American Psychiatric Association, 2013). Similar deficits are also observed in children and adults with ID (Sigafoos et al., 2017). Although *Drosophila* is a simple model, complex social interactions exist. Classically, fly sociability has been studied in the context of mating and aggression, by studying courtship behavior (Dockendorff et al., 2002; Villella and Hall, 2008) and male social dominance (Zwarts et al., 2012), respectively. Whereas the concept of sociability in these contexts substantially differs from human behaviors in this domain, new paradigms explore other, potentially more translatable, types of social behaviors, mostly based on inter-fly distance, and some have begun to be applied to ID/ASD genes.

One of the first approaches to characterize social interactions of *Drosophila* ID/ASD models was in Fragile X syndrome (FXS; Box 1), which showed that *dFMR1* mutant flies spend less time interacting with another fly in a neighboring chamber (divided by a mesh) (Bolduc et al., 2010). In a novel assay evaluating group formation, *Dnlg-2*-deficient flies showed decreased social interaction, whereas, in *Dnlg-4-*deficient flies, group formation was enhanced, implicating different members of the ID/ASD-associated Neuroligin family into opposite regulation of this social behavior (Corthals et al., 2017). *Dnlg-2* mutants also showed courtship and aggression deficits, implicating this gene in further aspects of social behavior (Hahn et al., 2013). Another assay with emerging relevance to ID/ASD assesses social space, the average distance in which flies position themselves relative to each other (Simon et al., 2012). Social space was increased in *rg* mutants, the ortholog of human *NBEA*, supporting it as an ASD-candidate gene (Wise et al., 2015). Social space was also affected in *FoxP*-null and pan-neuronal knockdown flies (Castells-Nobau et al., 2019). Interestingly, social space positively correlates with paternal and maternal age (Brenman-Suttner et al., 2018). As advanced paternal age at conception has been strongly linked with increased risk to ASD and other neuropsychiatric conditions due to increased rates of *de novo* mutations (Janecka et al., 2017; Sandin et al., 2016), it will be interesting to determine whether similar mechanisms underlie the *Drosophila* phenomenon.

Habituation, a form of non-associative learning, represents a selective filter through which an organism learns to ignore (and stops to react to) a familiar irrelevant stimulus. This mechanism, highly conserved throughout the entire animal kingdom, is thought to prevent information overload and to allow focusing on the available cognitive resources on relevant matters (McDiarmid et al., 2017; Ramaswami, 2014). Habituation is a proxy for synaptic plasticity (Castellucci et al., 1970; Larkin et al., 2010; Weber et al., 2002) and represents an important prerequisite for higher cognitive functions (Colombo and Mitchell, 2009; Kavšek, 2004; McDiarmid et al., 2017; Ramaswami, 2014). ASD is characterized by defective cortical filtering of sensory stimuli and information overload, which manifests in hypersensitivities, an ‘intense world’ perception (Ramaswami, 2014; Sinha et al., 2014), and probably also contributes to social deficits and other hallmark features (Barron et al., 2017; Kleinhans et al., 2009). A number of studies reported defective habituation in idiopathic ASD (Dinstein et al., 2012; Ewbank et al., 2015; Kleinhans et al., 2009; Pellicano et al., 2013). Habituation deficits have also been demonstrated in patients with FXS and in its mouse model (Restivo et al., 2005), as well as in a number of other ID/ASD mouse, zebrafish and fly models (Bariselli et al., 2018; Stessman et al., 2017; Wolman et al., 2014). Different types of habituation have been described in *Drosophila*, and a variety of assays are available for their assessment (Asztalos et al., 2007; Das et al., 2011; Kuntz et al., 2012; Paranjpe et al., 2012). Recently, *Drosophila* knockdown models of ∼300 ID genes were investigated in the light-off jump habituation paradigm (Box 1), revealing habituation deficits in more than 100 models (Fenckova et al., 2018). Interestingly, among the habituation-defective ID models, those with comorbid ASD were particularly enriched, suggesting that habituation could be a widely applicable readout for *Drosophila* studies of both disorders. Although habituation appears to exhibit strong face- and construct-validity, important prerequisites for accurate disease-modeling (Hmeljak and Justice, 2019), the predictive value of fly models for human habituation levels, and for ID and ASD clinical features, remains to be further characterized.

The above-discussed and other available assays and systems provide a rich repertoire to study the disease mechanisms of ID and ASD; they already made important contributions that significantly improve our understanding of the genetics and biology underlying specific aspects of neuronal morphology, function and behavior. In addition to the examples highlighted above, others have been previously featured in other reviews (Androschuk et al., 2015; Bolduc and Tully, 2009; van der Voet et al., 2014). With this large repertoire, *Drosophila* is a very powerful model that allows researchers to work across these different levels to accelerate fundamental and translational research for ID and ASD disorders.

## From fundamental gene function insights towards molecular networks and translational application

### Fragile X syndrome: from molecular mechanisms and novel functions to clinical trials

FXS is the most frequent and best-studied cause of monogenic ID and ASD (de Vries et al., 1997). It arises from a CGG-trinucleotide expansion and subsequent transcriptional silencing of the *FMR1* gene (Verkerk et al., 1991). The characteristic low IQ is highly comorbid with ASD traits, with a prevalence as high as 50% (Abbeduto et al., 2014). FXS has always been the forerunner in research for both disorders, in humans and other systems, including *Drosophila*. This is reflected by numerous discoveries in *Drosophila*, from abnormal synaptic architecture to learning and memory deficits (Bolduc et al., 2008; McBride et al., 2005; Sudhakaran et al., 2014; Zhang et al., 2001). The pathophysiological mechanisms underlying FXS and the contribution of *Drosophila* to this knowledge have been extensively discussed in dedicated reviews (De Rubeis et al., 2012; Drozd et al., 2018; McBride et al., 2013; Specchia et al., 2019). As illustrated by past work on FXS, *Drosophila* can be a useful tool to reveal changes in certain neurotransmitter systems, as now widely implicated in ID/ASD (Bear et al., 2004; Mariani et al., 2015; Muller et al., 2016). *Drosophila* provided the first pharmacological rescue of FXS-associated phenotypes, with mGluR antagonists that have been tested in clinical trials, unfortunately without success, as described and reviewed in detail elsewhere (Braat and Kooy, 2015; Chang et al., 2008; Duy and Budimirovic, 2017; McBride et al., 2005; Youssef et al., 2018). Decrease of the inhibitory neurotransmitter γ-aminobutyric acid (GABA) has also been intensively investigated in FXS *Drosophila* and other animal models (Gatto et al., 2014; Lozano et al., 2014). Importantly, the first and so far only unbiased large-scale *in vivo* drug screen for FXS, conducted in *Drosophila*, identified small molecules that interfere with both glutamatergic (excitatory) and GABAergic (inhibitory) signaling (Chang et al., 2008). Whereas for most ID/ASD genes it is still unknown in which neurons they act, it is obvious that this question can be efficiently addressed in *Drosophila* with its versatile genetic tools. Such knowledge is relevant to the development of treatment strategies for FXS and other ID/ASD disorders.

Also for FXS research, *Drosophila* continues to reveal aspects that may hint at treatment options, including those that could be relevant more widely to ID/ASD disorders. One aspect of FXS that has classically been rather overlooked is metabolic dysfunction (Bailey et al., 2010; Berry-Kravis et al., 2015; de Vries et al., 1993). A key regulator of metabolism in mammals and invertebrates, insulin signaling, was increased in the FXS *Drosophila* model (Monyak et al., 2017), along with deregulation of both carbohydrate and lipid metabolism (Weisz et al., 2018). This increase in insulin signaling in *dfmr1* mutants was shown to underlie the circadian defect of these flies, which could be rescued by either restoring *dfmr1* expression in the insulin-producing cells of the fly brain or by reducing the signaling pathway. Moreover, the enhanced insulin signaling also led to memory deficits (Monyak et al., 2017). Interestingly, pharmacological downregulation of insulin signaling with metformin also rescued the memory defects in *dfmr1* mutants. Similar findings have subsequently been reported in the FXS mouse model (Dy et al., 2018), and a controlled clinical trial has been recommended. Of note, the influence of metabolic state on cognition has been shown in both in flies and mammals (Chambers et al., 2015; Dou et al., 2005; Hirano et al., 2013; Placais and Preat, 2013). Since metabolic homeostasis is affected in a number of ID patients and models (Blanchet et al., 2017; Dunkley et al., 2017; Hsieh et al., 2014; Lin et al., 2010; Zheng et al., 2017), these findings provide avenues for developing innovative therapeutic approaches.

### ID/ASD genes cooperate in molecular networks: the EHMT1 module example

Another disorder for which *Drosophila* contributed much of our current knowledge is Kleefstra syndrome. The disorder, caused by haploinsufficiency of the eukaryotic histone methyltransferase 1 gene (*EHMT1*) (Kleefstra et al., 2010; Vermeulen et al., 2017), is characterized by ID, comorbid ASD in all patients reported so far (Vermeulen et al., 2017), behavioral problems and other clinical features, including recurrent infections and obesity (Kleefstra et al., 2012). Loss of *Drosophila G9a*, the ortholog of *EHMT1*, only resulted in subtle anomalies of da neurons and did not show other detectable nervous system architecture anomalies (Kramer et al., 2011). Nevertheless, *G9a* deficiency resulted in dramatic defects in courtship memory and light-off jump habituation caused by epigenetic changes in a set of target genes that featured the majority of known learning and memory genes. Interestingly, courtship memory could be restored by *G9a* re-expression in adulthood (Kramer et al., 2011), adding Kleefstra syndrome to a growing list of potentially reversible ID/ASD disorders.

Apart from learning and memory genes, G9a ChIP-seq data also revealed marked enrichment of genes implicated in immune defense and stress responses. Subsequent studies confirmed these results: *G9a* mutants were susceptible to virus infection (Merkling et al., 2015) and oxidative stress, the latter being caused by metabolic dysregulation (An et al., 2017; Riahi et al., 2019). This work identified energy availability as a generally limiting factor for oxidative stress resistance and further adds to metabolic dysregulation as a wider theme in ID/ASD.

G9a-related *Drosophila* work also makes a compelling case for the utility of this model organism in diagnostics, for what could be referred to as a ‘bedside-to-bench-and-back’ approach. In a cohort of patients with Kleefstra-syndrome-like appearance but no *EHMT1* mutations, next-generation sequencing approaches revealed single *de novo* mutations in five novel candidate genes (*MBD5*, *SMARCB1*, *KMT2C*, *NR1I3* and *MTMR9*) in four patients (Kleefstra et al., 2012). Testing pairwise genetic interactions with *G9a*, Kleefstra et al. showed that *KMT2C*, *MBD5*, *SMARCB1* and *NR1I3* genetically interact with EHMT1, uncovering an EHMT1-associated chromatin remodeling module of both synergistic and antagonistic interactions (Kleefstra et al., 2012). Notably, the fifth candidate gene, *MTMR9*, which was co-mutated in the patient with an *NR1I3* mutation, did not show any genetic interaction. This work strengthened *NR1I3* as the gene underlying the Kleefstra-syndrome-like phenotype in this specific patient, and enabled the genetic diagnosis of all four investigated patients.

Another study that further investigated the molecular pathology/transcriptional dysregulation common to *EHMT1* and *KMT2C* mutations found significant overlap in misregulated downstream target genes of the *Drosophila EHMT1* and *KMT2C* orthologs (*G9a* and *trr*) (Koemans et al., 2017a). One of the few direct target genes, dysregulated in both mutants, was the *Drosophila* ortholog of *Arc* (*Arc1*) (Koemans et al., 2017a), which also emerged as a relevant *EHMT1* target in recent mouse studies into Kleefstra syndrome (Benevento et al., 2016). *Arc* is an important neuron-specific regulator orchestrating multiple aspects of synaptic plasticity (reviewed in Shepherd and Bear, 2011), learning and memory (Whitlock et al., 2006). Interestingly, *Arc* had been previously linked to ID/ASD, both in the context of FXS (Krueger et al., 2011; Park et al., 2008; Yan et al., 2018) and Angelman syndrome (Box 1) (Greer et al., 2010; Kuhnle et al., 2013), suggesting convergent mechanisms between multiple ID/ASD disorders. Excitingly, *Drosophila* was key in groundbreaking work on the *Arc* mode of action (Ashley et al., 2018). The *Drosophila* Arc1 protein was shown to bind its own RNA *in vivo* and assemble into retrovirus-like capsids that are transferred in extracellular vesicles from the presynaptic NMJ terminal to its postsynaptic compartment. Abrogation of this process disrupted synaptic plasticity, uncovering a fundamentally new mechanism of synaptic communication (Ashley et al., 2018). A parallel study reported similar results in mice (Pastuzyn et al., 2018). Together, these examples highlight the relevance of findings in *Drosophila* for both fundamental and translational ID/ASD research.

## Future outlook

**Fig. 2.**
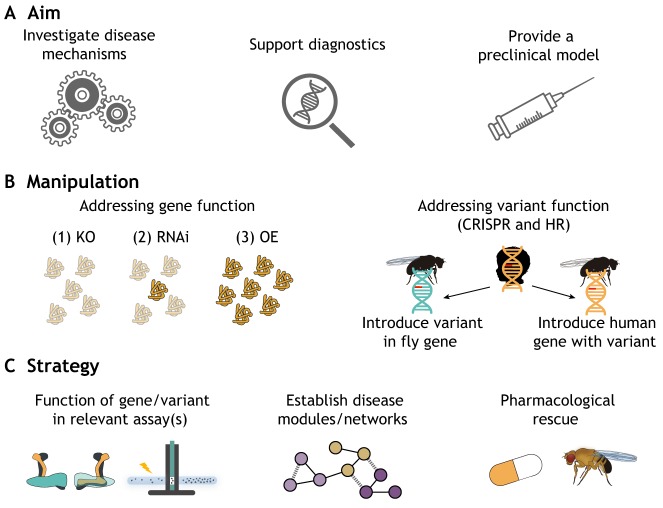
**Main challenges and applications of *Drosophila* as a model in future medical genomics for ID/ASD disorders.** (A) *Drosophila* research into ID and ASD can facilitate various aims, from dissection of disease mechanisms to shedding light onto pathogenicity of variants/mutations identified in the clinic, to providing preclinical models to assess the potential of treatment strategies. (B) Different genetic manipulations can be performed to target an ID/ASD gene of interest. Left: the most widely used manipulations to address gene function are: (1) complete ablation of proteins by gene knockout (KO), (2) decreased protein levels via RNA interference (RNAi)-mediated knockdown, or (3) increased protein levels via overexpression (OE) of the gene of interest. Right: the function of genetic variants can be addressed by either introducing the human variant [at the corresponding residue(s)] into the fly gene or by introducing the whole human gene with its variant in the fly genome. Both approaches can be realized using CRISPR/Cas9 (CRISPR) or homologous recombination (HR). (C) Several strategies can be followed to achieve the aims stated above, from assessing gene/variant function in ID/ASD-relevant assays (Fig. 1), establishing disease networks, to generating preclinical models, e.g. for pharmacological rescue.

As the above examples illustrate, *Drosophila* has made important contributions to our understanding of molecular mechanisms underlying ID/ASD disorders in the past decade. With the available resources and technologies, *Drosophila* is set to continue to contribute fundamental insights to this important field, and serve the great need for efficient and effective model organisms in translational research. Complementary to recent progress in uncovering ID and ASD genetics, *Drosophila* bears potential to push the boundaries of this field's main challenges by: (1) generating a better conceptual understanding of the pathophysiology of these disorders, (2) facilitating diagnostics, and (3) serving as a preclinical model for testing drugs and other treatment strategies (Fig. 2A). This final section further discusses how *Drosophila* can be exploited on all these fronts, and the important milestones and limitations of this endeavor.

### Strategies and opportunities for *Drosophila* disease modeling to overcome current bottlenecks

Unquestionably, future *Drosophila* work on ID/ASD-associated genes will also be based on manipulating the expression of their *Drosophila* orthologs through classical approaches. This includes the generation of knockout animals by various techniques, transgenic knockdown and/or overexpression (Fig. 2B), depending on the established or presumptive effect of the human disease alleles and on the further approach to be taken. Beyond addressing gene function, different studies have also investigated the effect of specific gene mutations by expressing these either in wild-type (Wan et al., 2000) or null/mutant backgrounds (Wu et al., 2015; Zamurrad et al., 2018), and comparing them to the effect of the non-mutated proteins. For such attempts, either transgenes expressing the human mutant proteins, or transgenes expressing the *Drosophila* genes with engineered, analogous mutations, can be used. Alternatively, gene replacement by homologous recombination and CRISPR/Cas9 genome-editing approaches now allow manipulation of the fly gene at its endogenous locus (de Brouwer et al., 2018; Mariappa et al., 2018) (Fig. 2B).

To evaluate the effect of specific mutations is not only of fundamental interest; it may well be that patients carrying different mutations also require different interventions, as most obvious for loss- versus gain-of-function mutations that likely require opposite manipulation. Furthermore, in the era of diagnostic exome sequencing in ID and ASD, the interpretation of genetic variants of unknown significance has become the major challenge in diagnostics (Di Resta et al., 2018). We can safely assume that the resulting need for functional investigation will further increase, at least in cases where human genetics/genomics fail to detect the same mutation in additional patients with similar phenotypes (van der Voet et al., 2014).

### Need for speed!

Extraordinarily efficient models are required to meet the current challenges, particularly in diagnostics, where the generation of relevant information is required in a rather short time and on demand. *Drosophila* already is in a pole position in this respect. Furthermore, we expect that *Drosophila* disease modeling will continue to benefit from the ever-increasing pool of readily usable resources of mutants, and from increasingly efficient phenotyping approaches. To date, large-scale resources for genetic manipulation, such as gene-disrupting P-element collections and libraries to induce conditional RNA interference or overexpression, exist. These allow researchers to manipulate the majority of genes in the *Drosophila* genome (Bellen et al., 2011; Bischof et al., 2013; Dietzl et al., 2007; Perkins et al., 2015), and thus also any evolutionarily conserved, established or newly identified, ID/ASD gene. A recent achievement that accelerates testing variants by rescue approaches is gene targeting with CRISPR-mediated integration cassettes (CRIMICs), which can be converted to T2A-Gal4 (or Trojan Gal4; Box 1) lines (Diao et al., 2015; Lee et al., 2018). A library of >1000 mutant T2A lines is already available (Lee et al., 2018), and genes can be nominated for CRIMIC generation via the webpage http://flypush.imgen.bcm.tmc.edu/pscreen/crimic/crimic-technique.html. The technology has been applied in a first study to demonstrate that *de novo* variants in the *EBF3* gene found in three individuals with ID are deleterious (Chao et al., 2017a).

Phenotypic characterization, particularly large-scale, remains laborious and often limited by data analysis and quantification processes. We discussed specific setups that facilitate data acquisition in the above-discussed disease-relevant paradigms. Other recent examples include the Fiji/ImageJ macro NMJ morphometrics to quantify morphological parameters in high throughput (Castells-Nobau et al., 2017; Nijhof et al., 2016). In behavioral research, several tools have been developed to assess and quantify learning and memory through courtship conditioning behavior, although their implementation appears to require programming or other skills to get operational (Dankert, 2009; Reza, 2013; Schneider, 2014). However, the assay can be efficiently conducted (Koemans et al., 2017b). Liu and colleagues developed a novel tracking and analysis pipeline that allows a large number of flies to be followed, and their social network quantified (Liu et al., 2018). One step further, the Janelia Automatic Animal Behavior Annotator (JAABA) is a machine-learning-based system to automatically track and quantify a wide variety of pre-defined behaviors (e.g. walking, touching, righting, etc.), and provides the computational framework for the quantification of additional behaviors of interest (Kabra et al., 2013). Further development of open-source setups and software for (semi)automated assessment and analyses of quantitative biological data can greatly contribute to the future success of *Drosophila* as a versatile disease model.

### Challenge 1: towards a conceptual understanding of the pathophysiology of these disorders

Reaching a higher throughput in the characterization of ID/ASD genes does not only increase data quantity, but also its quality. Based on shared phenotypes, gene modules that operate together can be recognized, with implications for fundamental (i.e. recognition of key pathways) and translational (i.e. the potential to target multiple ID/ASD models/disorders with the same treatment) research. So far, only a few large-scale studies into monogenic ID/ASD disorders have been conducted. These studies have implicated dozens of novel genes in neurotransmission and/or learning, and revealed neuronal substrates underlying the latter. Moreover, they uncovered functional modules that can predict additional phenotypes and demonstrated that ID genes associated with similar phenotypes in *Drosophila* are also associated with significant phenotypic similarity in humans (Fenckova et al., 2018 preprint; Kochinke et al., 2016; Oortveld et al., 2013).

Increasing the throughput of assays will also allow the transition from identifying monogenic to genetically more complex causes of ID/ASD. Two studies dissected phenotypes and genetic interactions among the *Drosophila* orthologs of genes co-affected by ID/ASD-associated copy number variations (CNVs). They tested pairwise interactions between conserved genes in both CNVs, and used readouts from cellular to behavioral systems (Grice et al., 2015; Iyer et al., 2018). Both studies identified extensive genetic interactions among the genes located in a single CNV locus and beyond, and proposed that variants in multiple genes contribute to the respective disease phenotypes.

To our knowledge, no studies systematically mined public genome-wide *Drosophila* data to identify characteristic phenotypes or patterns associated with *Drosophila* ID/ASD orthologs. *Drosophila* can further contribute to the identification of common phenotypes and mechanisms underlying ID/ASD in the future.

### Challenge 2: towards *Drosophila* as a tool in diagnostics

As discussed, the need for systems that can inform medical genomics about the causal relationship between a mutation and a clinical phenotype is enormous. For ID/ASD, *Drosophila* researchers have so far taken two approaches. First, they investigated whether manipulating the expression of a candidate gene can cause an ID/ASD-relevant phenotype in flies, providing support for such a causal relationship. Second, they addressed whether an identified mutation affects gene function, even if this does not (or not obviously) relate to the clinical phenotype. Both approaches have value; ideally, future studies will combine testing patient-specific mutations with an assay tailored to the clinical phenotype (Fig. 2B,C). In addition, the genetic interaction/network approaches with known disease genes can be exploited where one or more genes have already been implicated in a specific syndrome (Fig. 2C).

To facilitate the use of *Drosophila* in diagnostics, it is not only important to generate disease-relevant data in this organism, but also to organize them in a way that they can be accessible across disciplines. However, major barriers in the communication between clinicians and fundamental *Drosophila* researchers often hinder the development of effective interdisciplinary collaborations (Chao et al., 2017b). These pitfalls, as well as the initiatives, resources and tools for clinicians and researchers to facilitate effective bi-directional dialogues, have been discussed in detail elsewhere (Chao et al., 2017b; Şentürk and Bellen, 2018; Yamamoto et al., 2014). Open-access databases – such as MARRVEL, which integrates data from human disease research to biochemical data and that from multiple model organisms (Wang et al., 2017); FlyBase [http://flybase.org (Gramates et al., 2017)], with its implemented Human Disease Model section (Millburn et al., 2016); and the Monarch Initiative, connecting genotypes to phenotypes across species (Mungall et al., 2017) – are at least a start to increasing interspecies research collaborations. A series of recent papers in the ID/ASD field that combine clinical and *Drosophila* data with the identification of genetic defects in patients argue that clinicians, and human and *Drosophila* geneticists nowadays find each other more efficiently (de Brouwer et al., 2018; Fattahi et al., 2018; Gonçalves et al., 2018; Koemans et al., 2017a; Nixon et al., 2019; Straub et al., 2018).

A persistent limitation to the implementation of *Drosophila* in diagnostics is its evolutionary distance from humans. A quarter of all human genes do not have a *Drosophila* counterpart, and a significant amount of human coding variants will not affect conserved residues. The former will, in many cases, also limit the fly's value to point to causal variants or genes among multiple ones affected in a patient (i.e. by a CNV or by multiple *de novo* mutations); if some variants cannot be modeled, the outcome of such experiments will remain incomplete.

### Challenge 3: towards successful treatment strategies

Research in one or even across different animal models has demonstrated that the cognitive defects in some ID/ASD disorders, such as FXS, neurofibromatosis type 1 and Kleefstra syndrome, may be reversible in adulthood (Kramer et al., 2011; Lee et al., 2014; McBride et al., 2005). *Drosophila* could readily be used to assert reversibility for dozens to hundreds of uncharacterized ID/ASD genes with the same approach. Such disorders could then be prioritized for intervention.

While FXS appeared as a success story in translational medicine for some years, so far clinical trials have failed. Despite the progress, our treatment options for ID/ASD remain limited. How can we improve in the future? Intervention strategies that were successful in *Drosophila* will need confirmation in other systems and, if positive, to be tested in clinical trials. One still unexplored, conceptually novel approach in ID/ASD drug identification would be to use high-throughput amenable cognitive readouts (i.e. learning or memory paradigms) for large-scale drug screening in ID/ASD. The identified compounds would eventually need to be tested in higher organisms and prove their utility in patients.

## Conclusions

A number of major challenges in ID and ASD research lie ahead. *Drosophila*, with its unique resources and advantages, may be one of the organisms that is best equipped to meet many of the current bottlenecks limiting the translation of successful preclinical research to clinical application. Importantly, the community needs not only this model, but also research funding and training to raise the next generation of creative interdisciplinary scientist who will take up this translational endeavor.
